# Malignant Insulinoma: Diagnostic Difficulties and Treatment Strategies in a Case of Persistent Hypoglycemia

**DOI:** 10.7759/cureus.74700

**Published:** 2024-11-28

**Authors:** Shubh Mehta, Ahan Banker, Jay V Shah

**Affiliations:** 1 Department of Internal Medicine, B. J. Medical College and Civil Hospital, Ahmedabad, IND

**Keywords:** hypoglycemia, malignant insulinoma, pancreatic insulinoma, pancreatic neuroendocrine tumor (pnet), whipple's triad

## Abstract

Hypoglycemia in non-diabetic individuals is a rare but critical condition that often signals an underlying pathology. Insulinoma, a rare neuroendocrine tumor of the pancreas, is a key differential diagnosis. As the most common functional pancreatic neuroendocrine tumors, insulinomas originate from pancreatic islet cells and are predominantly benign. However, malignant cases, although rare, represent a significant diagnostic challenge. Here, we report a 67-year-old female with recurrent hypoglycemic episodes presenting as diaphoresis and palpitations, alleviated by glucose intake. Initial tests revealed critically low blood sugar levels and elevated fasting insulin and C-peptide. Despite a normal abdominal CT, a PET-CT scan identified an exophytic pancreatic lesion with metastases to retroperitoneal lymph nodes, and malignancy was confirmed by histopathological examination. This case highlights the necessity of considering insulinoma in the differential diagnosis of unexplained hypoglycemia. Accurate and timely diagnosis, along with appropriate imaging and management, is crucial for effective treatment and improved patient outcomes.

## Introduction

Hypoglycemia is a critical condition that requires prompt diagnosis and treatment, particularly in non-diabetic individuals where its occurrence is uncommon and often indicative of an underlying pathology. One of the key differential diagnoses in such cases is insulinoma, a rare neuroendocrine tumor of the pancreas.

Insulinoma is the most common pancreatic functional pancreatic neuroendocrine tumor (NET), derived from pancreatic multipotent stem cells or neuroendocrine islet cells that secrete insulin [1]. Insulinomas are most commonly benign, well-differentiated NETs, whereas malignant neoplasms account for 5.8% of cases [2]. The incidence of insulinoma is around one to four per million per year representing 1-2% of pancreatic tumors [2,3]. The symptoms of hypoglycemia include sympathetic overactivity symptoms, manifesting as fatigue, tremor, weakness, sweating and tachycardia, and neuroglycopenic symptoms resulting in central nervous system (CNS) disturbances such as confusion, disorientation, stupor, blurring of vision, delirium, coma or convulsions [4,5]. Whipple's triad is the diagnostic criterion for insulinoma, including symptoms of hypoglycemia, documented low blood glucose (40-50 mg/dL), and relief of symptoms post intravenous administration of glucose [5].

This case report presents a 67-year-old female with recurrent episodes of hypoglycemia, ultimately diagnosed with a malignant insulinoma. This case underscores the diagnostic challenges and the importance of including insulinoma in the differential diagnosis of unexplained hypoglycemia in the Indian demography.

## Case presentation

A 67-year-old female presented with a three-month history of recurrent diaphoresis and palpitations. Her symptoms were alleviated momentarily by consuming sweet food items such as candy or sugar cubes. 

**Figure 1:**
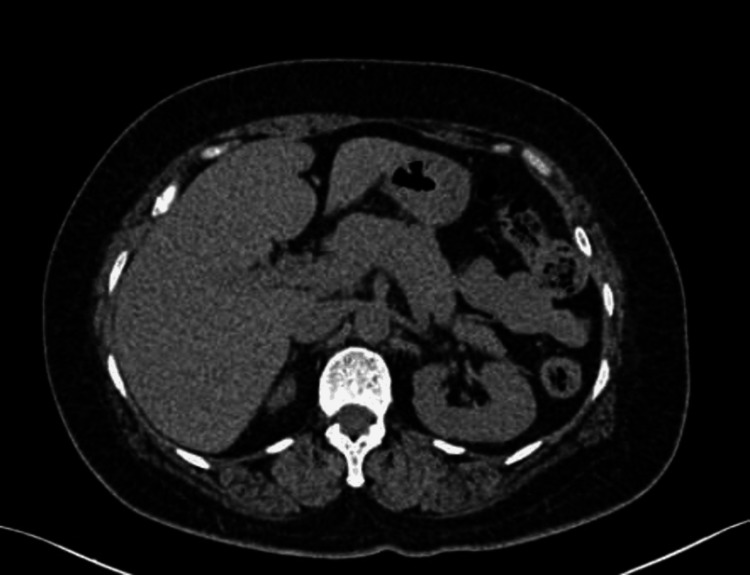
Normal abdominal computed tomography (CT)

On arrival at the Emergency Department, her random blood sugar (RBS) was critically low at 37 mg/dl. She was immediately treated with three pints of a D25 solution and continued on a D5 infusion. Further investigations revealed elevated fasting insulin levels at 45 µU/mL and a C-peptide level of 2.8 ng/mL with an RBS of 42 mg/dl. Despite these findings, a CECT scan of the abdomen was normal (Figure 1), and the patient’s HbA1c was 4.6%, indicating no chronic glycemic control issues. 

Given the clinical suspicion of insulinoma, she was started on octreotide 100 µg subcutaneously every eight hours. This treatment led to a cessation of the need for a D25 infusion. Following initial management, the patient was transitioned to oral diazoxide 25 mg three times a day before meals. 

**Figure 2:**
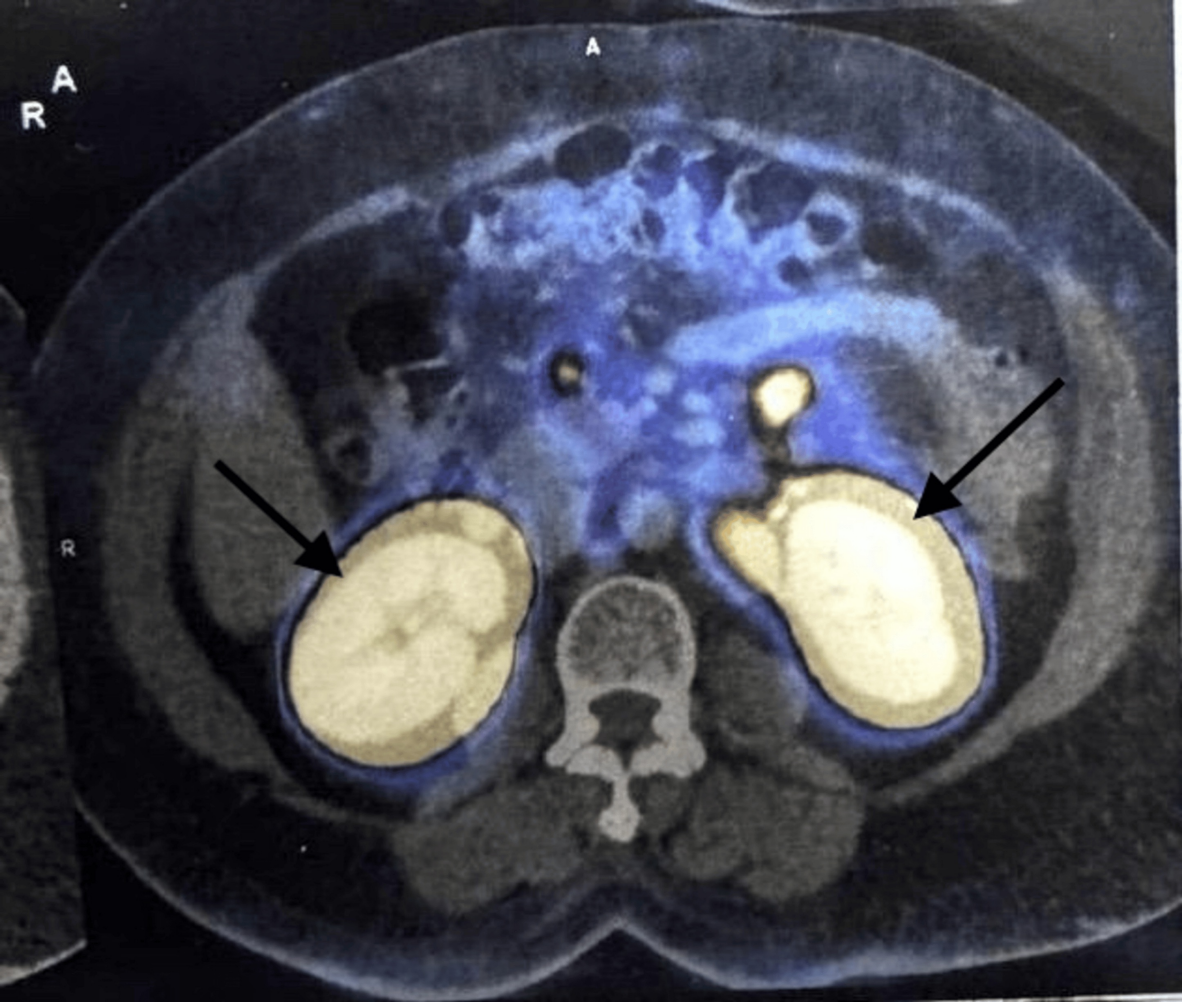
Ga-68 DOTA-exendin positron emission tomography-computed tomography (PET-CT) with a tracer avid exophytic lesion involving the neck of the pancreas

To confirm the diagnosis, a whole-body PET-exendin scan was performed. Ga-68 DOTA-exendin (3.5 mCi) was injected IV followed by whole-body PET-CT images at one hour (Figure 2). A poorly defined, tracer-avid, predominantly exophytic lesion involving the neck of the pancreas was noted, which appeared to be neoplastic. A few heterogeneous, tracer-enhancing avid nodes were also seen involving left supraclavicular, peripancreatic, portocaval, aortocaval, pre-paraaortic, and mesenteric regions. The largest one was seen in the paraaortic region measuring 2.3 x 3.2 cm revealing an intense tracer uptake. 

**Figure 3:**
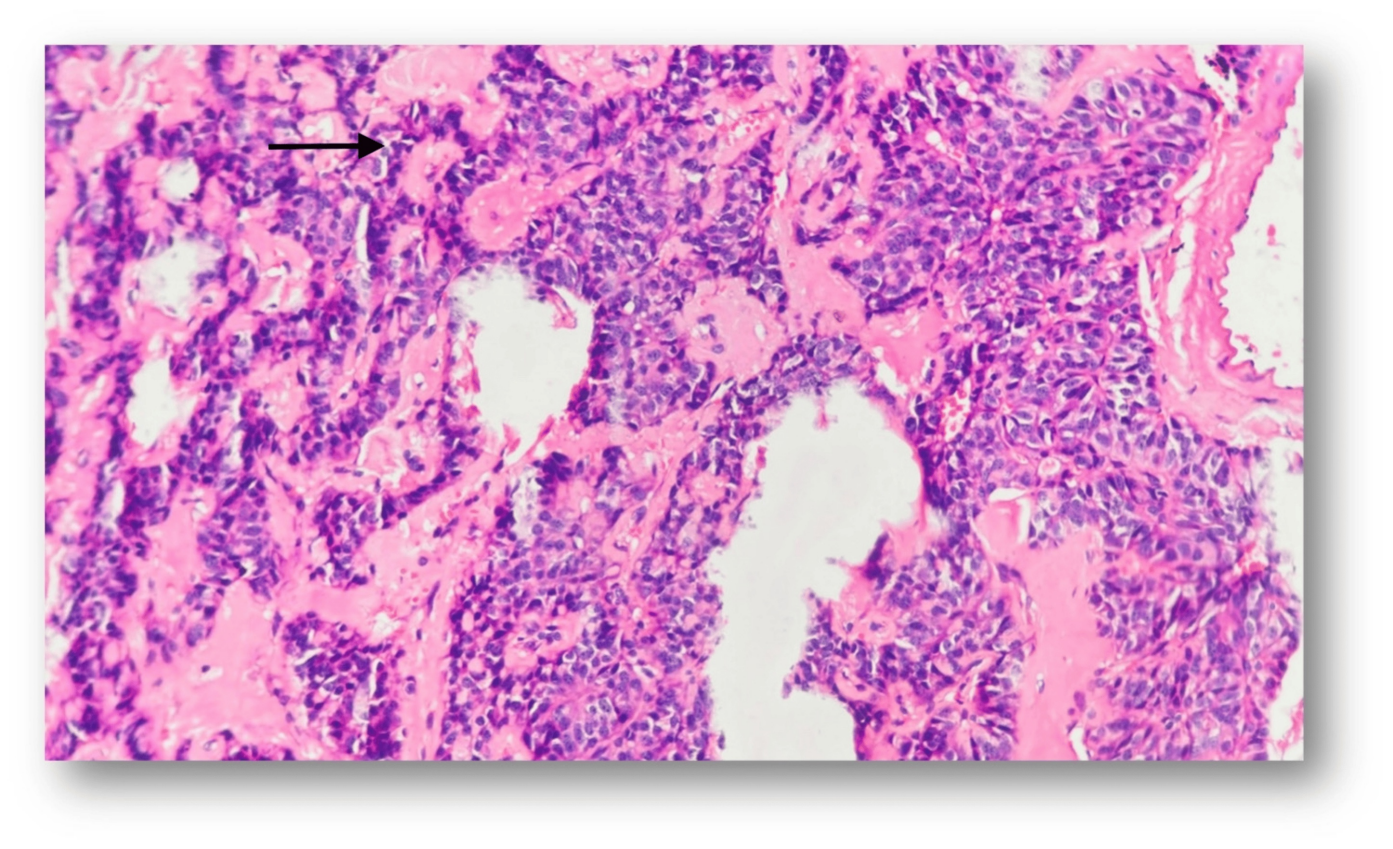
Histopathology showing well-differentiated neuroendocrine tumor.

This diagnosis was subsequently confirmed through histopathological examination (HPE) (Figure 3). Pancreatic and retro-peritoneal lymph node specimens were taken. A well-differentiated neuroendocrine tumor of grade 1/2 with metastasis to retroperitoneal lymph nodes was found (TNM staging - pT1(m)N1). 

The patient had no history of sulfonylurea use, and all other laboratory reports were within normal limits. 

The diagnostic and therapeutic approach successfully managed her hypoglycaemic episodes and confirmed the presence of a malignant insulinoma, highlighting the importance of prompt and accurate diagnosis in managing such endocrine disorders.

## Discussion

Insulinoma is a rare neuroendocrine tumor, its origin being the pancreatic beta cells. While they are primarily benign, malignant cases are observed, as we have largely associated with elevated insulin and proinsulin levels along with a size larger than that of benign tumors [6]. Most cases arise spontaneously and are sporadic in nature, while familial cases are associated with MEN-1 syndrome, characterized by a predisposition to multiple endocrine tumors such as pituitary and parathyroid adenomas in addition to pancreatic tumors [7].

Insulinomas primarily present with hypoglycemia, neuroglycopenia, and symptoms related to catecholamine excess, released as a result of hypoglycemia (Table 1) [8].

**Table 1:** Manifestations of insulinomas. Adapted from [8].

Symptoms	Number of patients	Percentage
Weakness	59	88
Sweating	58	87
Palpitations	57	85
Coma	49	73
Behavioral problems	47	70
Seizures	33	50

The classic triad of symptoms, known as Whipple’s triad, consists of symptoms of hypoglycemia, documented low blood glucose (40-50 mg/dL), and relief of symptoms after the intravenous administration of glucose [5]. Our patient showcased all three criteria of Whipple’s triad. The other parameters studied are mentioned in Table 2.

**Table 2:** Investigation results

Parameter	Patient's value	Units	Reference range
Random blood sugar (RBS)	37	mg/dL	70-140 mg/dL
C-peptide at RBS of 42 mg/dL	2.8	ng/mL	0.9-7.1 ng/mL
Fasting insulin	45	µU/mL	2-25 µU/mL
HbA1c	4.6%	%	4.0-5.6%
CECT scan	Normal	-	No abnormality
Proinsulin levels	25	pmol/L	<5 pmol/L
Beta-hydroxybutyrate	0.1	mmol/L	0.02-0.27 mmol/L
Glucose tolerance test (two-hour)	45	mg/dL	<140 mg/dL
Sulfonylurea screen	Negative	-	Negative
72-hour fasting test (insulin)	48	µU/mL	<3 µU/mL (in hypoglycemia)
Plasma insulin-to-glucose ratio	0.8	-	<0.3
Insulin antibody test	Negative	-	Negative
Chromogranin A (CgA)	110	ng/mL	<100 ng/mL
Endoscopic ultrasound (EUS)	Lesion detected	-	Normal/lesion detected
MRI or CT scan	Lesion detected	-	Normal/lesion detected
Arterial stimulation with venous sampling (ASVS)	Positive for insulin secretion	-	-

Insulinomas can also present with recurrent hypoglycemic seizures, which are corrected after localizing and resecting the lesion [9].

Insulinomas are localized using either of the following modalities: abdominal CT, endoscopic ultrasound, or magnetic resonance imaging with dynamic sequences [10]. Traditional imaging modalities like CT and MRI have shown limited sensitivity in detecting small or atypically located insulinomas, often leading to diagnostic challenges. Studies report that modalities such as endoscopic ultrasound (EUS) and ^68Ga-DOTA-somatostatin analog PET/CT have improved accuracy but are invasive or require specialized expertise. Advanced functional imaging techniques like ^68Ga-NODAGA-exendin-4 PET/CT have demonstrated significantly higher sensitivity, localizing insulinomas in up to 94% of cases and succeeding when other methods fail. These advancements highlight the importance of integrating such tools into diagnostic protocols to enhance detection and guide surgical management [11].

The primary treatment of insulinoma is surgical excision with the aim of preserving pancreatic function [12]. Medical management with octreotide or diazoxide is utilized as preventive measures to avoid acute episodes, or when surgical management is contraindicated, or tumors are unresectable as is highlighted in our patient [9,12]. Verapamil can be prescribed in refractory cases due to its inhibiting action on insulin release [9].

Our patient presented with recurrent adrenergic symptoms. She was found to be hypoglycemic on further testing. Her abdominal CT was normal, but the PET-CT displayed an exophytic lesion involving the neck of the pancreas and distant lymph nodes as well. The histopathological examination resulted in a well-differentiated grade 1/2 neuroendocrine tumor, thus confirming the diagnosis of insulinoma with retroperitoneal lymph node metastasis. She was treated with octreotide and diazoxide.

## Conclusions

This case underscores the critical importance of considering insulinomas in the differential diagnosis of unexplained hypoglycemia, particularly in patients who meet Whipple's triad. Early recognition of this rare but treatable condition is essential to prevent potentially severe complications. Clinicians should maintain a high index of suspicion in individuals with recurrent neuroglycopenic and adrenergic symptoms. Moreover, when traditional imaging modalities fail to localize the tumor, the use of advanced imaging techniques, such as endoscopic ultrasound or specialized nuclear imaging, can significantly enhance diagnostic accuracy. Prompt diagnosis and precise localization enable timely surgical intervention, which is the definitive treatment and can dramatically improve patient outcomes.
